# AC Corrosion of Carbon Steel under Cathodic Protection Condition: Assessment, Criteria and Mechanism. A Review

**DOI:** 10.3390/ma13092158

**Published:** 2020-05-07

**Authors:** Andrea Brenna, Silvia Beretta, Marco Ormellese

**Affiliations:** Dipartimento di Chimica, Materiali e Ingegneria Chimica “Giulio Natta”, Politecnico di Milano, I-20131 Milan, Italy; silvia.beretta@polimi.it (S.B.); marco.ormellese@polimi.it (M.O.)

**Keywords:** alternating current, cathodic protection, carbon steel, pipeline, AC interference corrosion, AC corrosion assessment, protection criteria, corrosion mechanism

## Abstract

Cathodic protection (CP), in combination with an insulating coating, is a preventative system to control corrosion of buried carbon steel pipes. The corrosion protection of coating defects is achieved by means of a cathodic polarization below the protection potential, namely −0.85 V vs. CSE (CSE, copper-copper sulfate reference electrode) for carbon steel in aerated soil. The presence of alternating current (AC) interference, induced by high-voltage power lines (HVPL) or AC-electrified railways, may represent a corrosion threat for coated carbon steel structures, although the potential protection criterion is matched. Nowadays, the protection criteria in the presence of AC, as well as AC corrosion mechanisms in CP condition, are still controversial and discussed. This paper deals with a narrative literature review, which includes selected journal articles, conference proceedings and grey literature, on the assessment, acceptable criteria and corrosion mechanism of carbon steel structures in CP condition with AC interference. The study shows that the assessment of AC corrosion likelihood should be based on the measurement of AC and DC (direct current) related parameters, namely AC voltage, AC and DC densities and potential measurements. Threshold values of the mentioned parameters are discussed. Overprotection (*E*_IR-free_ < −1.2 V vs. CSE) is the most dangerous condition in the presence of AC: the combination of strong alkalization close to the coating defect due to the high CP current density and the action of AC interference provokes localized corrosion of carbon steel.

## 1. Introduction

Cathodic protection (CP), in combination with an insulating coating, is a well-known electrochemical technique that reduces (or halts) the external corrosion rate of buried carbon steel pipes used to transport liquid or gas. In CP condition, the corrosion rate is reduced below 0.01 mm·a^−1^, which is the maximum acceptable value fixed by CP standards [1,2]. Carbon steel in aerated soil, i.e., where oxygen reduction is the controlling cathodic process, operates in CP condition if the IR-free potential (excluding the ohmic drop contribution in soil) is more negative than −0.85 V vs. CSE (Cu/CuSO_4_ reference electrode, +0.318 V vs. standard hydrogen electrode, SHE) [1,2].

The presence of alternating current (AC) interference on buried pipelines in free corrosion or under CP condition can lead to severe localized corrosion through the pipe thickness. In the case of AC interference, the sources of electrical disturbance are the high-voltage power lines (HVPL) or the AC-electrified railways (fed by a high voltage line at 50 or 60 Hz), the receptor is the pipeline that runs parallel to the interference source and the coupling mechanism occurs mainly via a resistive (or conductive) and inductive (or electromagnetic) mechanism [3].

The resistive coupling is primarily a concern when there is a fault or an unbalanced condition on the power line and large currents and voltages are conveyed to the earth during the HVPL short circuit. Although the interference time is short, it represents a hazard to the operators and to the buried pipe corresponding to the coating defects. The inductive coupling occurs when AC flowing in phase wires produces an electromagnetic field inducing alternating currents and voltages to the pipeline, which shares the way with the power line. The induced AC voltage depends on the length of parallelism with the power line, and it is inversely proportional to the distance between the HVPL and the pipeline; the AC density, i.e., the current for unit surface, is a function of AC voltage, the coating defect dimension and soil resistivity.

Nowadays, there is agreement that corrosion induced by AC interference can occur even on carbon steel structures that fully respect the CP criterion and that AC corrosion is less than that provoked by the equivalent direct current, i.e., considering the same current density. In the presence of AC interference, the CP criteria reported by international standards [1,2] are not sufficient to prove that steel is protected from corrosion. In the past 50 years, great effort has been made in order to propose criteria to assess AC corrosion likelihood and to understand the mechanism by which AC causes corrosion. This effort brought about the international standard ISO 18086 (Corrosion of metals and alloys—Determination of AC corrosion—Protection criteria) [4] that replaced in 2017 the EN 15280 standard (Evaluation of a.c. corrosion likelihood of buried pipelines applicable to cathodically protected pipelines). The ISO 18086 standard provides monitoring procedures, mitigation measures and information to deal with long-term AC interference. Nevertheless, some aspects related to the phenomenon were not fully understood and the protection criteria as well as the corrosion mechanism have been debated for a long time.

This paper deals with a narrative literature review, which includes a deep analysis of the AC corrosion phenomenon, in particular the assessment of AC interference, and the evaluation of AC corrosion likelihood and interference levels, the corrosion mechanism.

## 2. Assessment of AC Corrosion Likelihood

The assessment of AC interference likelihood on a buried pipeline should include several parameters related to both the interference source and the interfered structure. During the design phase, the evaluation of AC interference on a buried structure can be carried out by mathematical/electrical modelling, e.g., according to EN 50443 (Effects of electromagnetic interference on pipelines caused by high voltage AC electric traction systems and/or high voltage AC power supply systems) [5] or IEEE Guide for Safety in AC Substation Grounding [6]. These approaches aim to evaluate the tolerable AC voltage based on parameters, such as the electrical configuration of the AC power line, the distance between the AC source (power line or traction system) and the pipeline, the insulation properties of the coating as well as soil resistivity. In the case of existing structures, field measurements can be used as an alternative to calculation. According to calculations or field measurements, relevant mitigation measures should be installed to decrease the AC corrosion probability. Nevertheless, not only electrical parameters are involved in the AC corrosion mechanism and an electrochemical approach is required for an understanding of the mechanism, in particular in the presence of cathodic protection.

According to ISO 18086 [4], the assessment of AC corrosion should be performed by evaluation of some or all of the following parameters:

- AC voltage, *V*_AC_;
- AC density, *i*_AC_;
- DC density, *i*_DC_;
- AC/DC densities ratio, *i*_AC_/*i*_DC_;
- DC potential (IR-free potential, *E*_IR-free_, and ON potential, *E*_ON_);
- Soil resistivity, *ρ*.

### 2.1. AC Voltage

The measurement of the AC voltage, *V*_AC_, on a pipeline is carried out with respect to a reference electrode located at remote position, i.e., where the AC voltage gradient does not change and is close to zero. The AC voltage gradient is measured by means of two-reference electrodes spaced 1 to 5 m transverse to the pipeline.

According to [4], acceptable AC voltages on the pipeline in CP condition are lower than 15 V r.m.s. measured as an average over a representative time (e.g., 24 h). According to NACE SP0177 (Mitigation of Alternating Current and Lightning Effects on Metallic Structures and Corrosion Control Systems) [7], the maximum AC voltage is set at 15 V with respect to local earth (approximately 1 m); this threshold is mostly driven by safety considerations (shock hazard).

In a recent work, Tang et al. [8] investigate the effects of several parameters on the electric field distribution of AC interference, such as the unbalanced current magnitude, soil and coating resistivity and the distance between the power line and the pipeline. By means of numerical simulation, the authors conclude that the reference electrode should be placed farther from the pipeline route with the increase of mitigation wire length, soil resistivity and the distance between the power line and the structure; conversely, the earth remote position is closer to the pipe by increasing coating resistivity if mitigation is applied [8].

### 2.2. AC Density

AC and DC densities on a coating defect control both the AC interference and the CP level, respectively. Contrary to the AC voltage measurement, the AC density, *i*_AC_, cannot be readily determined. The numerical approach considers the calculation of AC density from the AC voltage, the soil resistance, *ρ*, and the diameter, *ϕ*, of a circular coating defect, according to the following equation, as reported in [8,9,10,11,12]:

(1) $$i_{\mathrm{AC}}=\frac{8\cdot V_{\mathrm{AC}}}{\rho \cdot \pi \cdot \phi }$$

Considering a maximum AC voltage of 15 V measured on a circular coating defect of 1 cm^2^ and a medium soil resistivity of 100 Ω·m, the expected AC density threshold is about 30 A·m^−2^. Nevertheless, this calculation is generally not possible since the coating defect area is not known. Moreover, the application of CP can significantly change the electrolyte composition in proximity to the coating defect and consequently the local soil resistivity. The formula is valid when the coating defect size is larger than the coating thickness, although rigorous calculations are available [13]. The current density can only be estimated by means of coupons or probes. According to ISO 18086 [4], the measurement of AC density has to be carried out on a 1 cm^2^ coupon surface area connected to the structure.

**Figure 1:**
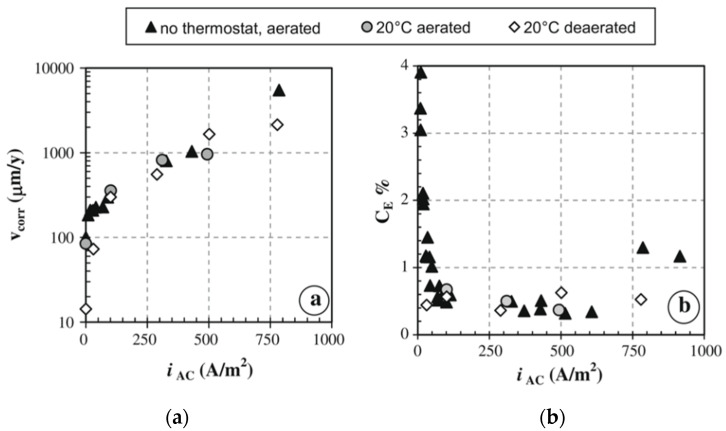
Weight loss tests on carbon steel in free corrosion condition exposed to a soil-simulating solution: (**a**) corrosion rates vs. AC density (*i*_AC_) and (**b**) AC corrosion efficiency vs. AC density (*i*_AC_) as reported by Goidanich et al. [16].

The definition of a critical threshold of AC density over which AC corrosion could occur is still controversial and large data variability is found. Compared to DC interference corrosion, AC corrosion of carbon steel is lower considering the same current density. Since the sixties of the last century [14,15], the effect of AC density was determined in terms of “equivalent DC density”, defined as the percent ratio of the weight loss caused by AC to the expected weight loss due to the same DC density. The values of equivalent DC density are in the range between 0.1% and 0.3% with AC density up to 600 A·m^−2^ [14,15]. Using laboratory tests on carbon steel in soil-simulating solution (1200 mg·dm^−3^ sulphates, 200 mg·dm^−3^ chlorides), Goidanich et al. [16] reported that AC corrosion efficiency (defined similarly to the “equivalent DC density”) is lower than 1% when AC density ranges from 50 to 500 A·m^−2^, but it increases up to 4% for AC density lower than 50 A·m^−2^ (Figure 1). In 2010, Fu and Cheng [17] reported comparable results.

Gummow et al. [18] stated that the corrosion rate increases with increased AC density greater than 20 A·m^−2^ and becomes significant at AC densities greater than 100 A·m^−2^, regardless of the magnitude of CP density. Based on laboratory tests, Pourbaix et al. [19] reported that AC corrosion is associated with the IR-free potential oscillation during interference but it is not related to a critical value of the AC density. As reported by Yunovich and Thompson [20], steel corrosion can significantly increase in the presence of 20 A·m^−2^ AC density: the measured corrosion rate at 20 A·m^−2^ AC density is nearly two times higher than that of the control specimen in free corrosion condition and decreases with the application of CP. This last consideration introduces the need of the additional consideration of the cathodic DC density.

### 2.3. AC/DC Current Density Ratio

For carbon steel structures under CP conditions, AC corrosion likelihood should be evaluated also considering the level of DC polarization, by means of the IR-free potential or DC density. The latter can be measured by means of a corrosion coupon or probes with a known surface area, e.g., 1 cm^2^. In order to assess AC corrosion conditions, it is better to refer to the AC density-DC density ratio (*i*_AC_*/i*_DC_), which is dimensionless. Nevertheless, use of only the *i*_AC_*/i*_DC_ ratio could be misleading in the assessment of AC corrosion likelihood, i.e., different AC corrosion conditions can be represented by the same *i*_AC_*/i*_DC_ ratio. For instance, an *i*_AC_*/i*_DC_ ratio equals to 10 results from an interference condition of 30 A·m^−2^ AC density in the presence of 3 A·m^−2^ DC density or 3 A·m^−2^ AC density with 0.3 A·m^−2^ DC density. Although the ratio between the current densities is equal in the two conditions, they represent a different corrosion risk, i.e., 3 A·m^−2^ AC density is not recognized as a threat, dissimilarly from 30 A·m^−2^. As discussed in Paragraph 3, several authors [21,22,23,24,25,26,27] investigated the effect of *i*_AC_*/i*_DC_ ratio on corrosion rate, proposing different threshold limits. ISO 18086 standard [4] reports that AC corrosion can be mitigated by maintaining the *i*_AC_*/i*_DC_ ratio less than 3 over a representative time (e.g., 24 h) and it is valid for DC density greater than 10 A·m^−2^ (severe over-protection condition) and AC density over 30 A·m^−2^.

### 2.4. AC Frequency

There is full agreement that the corrosion rate decreases by increasing the frequency of the AC signal. The effect of frequency has been investigated on mild steel, nickel and copper-nickel alloys [28,29,30,31,32,33,34,35]. AC can cause severe corrosion at the industrial frequencies of 50 or 60 Hz, while the effect decreases at frequencies higher than 150 Hz.

Fernandes et al. [28] and the other authors [29,30,31,32] proposed a kinetic interpretation: by increasing the frequency, the time between the anodic and cathodic half-cycles becomes shorter and the metallic ions dissolved during the anodic period would be available for the subsequent deposition in the cathodic cycle.

Guo et al. [34,35] reported that at an AC density of 50 A·m^−2^, the corrosion rate of X60 steel decreases from 1.2 mm·a^−1^ at 10 Hz frequency to about 0.6 mm·a^−1^ at 50 Hz. In parallel, the free corrosion potential increases to about 50 mV. Yunovich and Thompson [36] proposed an electrical circuit in order to simulate the behavior of a steel specimen exposed to soil varying the frequency of the AC signal. The model shows that the corrosion current in the circuit decreases with increasing frequency and is approximately 0.3% of the total current at 60 Hz frequency, in agreement with the results using weight loss tests, as reported in Figure 1.

### 2.5. Soil Resistivity and Chemical Composition

According to Equation (1), the AC density corresponding to a coating defect depends on the alternating voltage and on the spread resistance, which is the ohmic resistance through a coating defect (or a corrosion coupon) to earth. The ISO 18086 standard [4] reports an empirical relation between soil resistivity and AC corrosion risk:

- *ρ* < 25 Ω·m: very high risk;
- 25 Ω·m < *ρ* < 100 Ω·m: high risk;
- 100 Ω·m < *ρ* < 300 Ω·m: medium risk;
- *ρ* > 300 Ω·m: low risk.

Soil resistivity close to a coating defect is significantly affected by the electrochemical reactions at the metal-to-electrolyte interface, due to the application of the CP current. In CP condition, oxygen reduction (O_2_ + 2H_2_O + 4e^−^ → 4OH^−^) and, at lower potential, hydrogen evolution (2H^+^ + 2e^−^ → H_2_), cause a growth of alkalinity at the metal surface. The pH value can increase over 10 and up to 12–13 at very high cathodic current densities. The local soil chemical composition can play a crucial role in the AC corrosion assessment, as documented in [27,37]. Earth-alkaline ions (as Ca^2+^ and Mg^2+^), moved towards the metal surface by the CP electric field, form slightly soluble hydroxides; the pH increase, shifting the carbonate-bicarbonate chemical equilibrium, favors the growth of a scale of calcium and magnesium carbonate that increases the spread resistance. Otherwise, alkaline cations (as Na^+^, K^+^ or Li^+^) form not-scaling hydroxides. Büchler et al. [37] reported a reduction of AC density due to the growth of chalk layers on the surface in the presence of calcium ions.

Recently, Xiao et al. [27] reported that the spread resistance of a X70 steel specimen at constant CP potential and different AC densities is higher in the presence of calcium and magnesium ions. Moreover, the corrosion rate of the specimens exposed to higher content of Na^+^ was greater than that in the presence of earth-alkaline ions at the same potential and AC density (100 A·m^−2^, 300 A·m^−2^).

### 2.6. Effect on DC Potential (Free Corrosion Condition)

#### 2.6.1. Negative Shift of Potential

There is general agreement that the free corrosion potential of carbon steel, i.e., without cathodic protection, decreases as the AC density increases. This has been documented from the sixties of the last century. Bolzoni et al. [38] reported laboratory tests on the influence of AC interference on carbon steel corrosion in free corrosion condition in different environments (sulfate and chloride aqueous solutions, with or without oxygen, simulating soil conditions and seawater). AC was overlapped to the specimens ranging from 10 to 6000 A·m^−2^. The free corrosion potential of carbon steel in chloride and sulfate solutions decreases as AC density increases. At AC densities below 100 A·m^−2^, the DC potential variation was low (about 50 mV); above 100 A·m^−2^, the effect was higher (100–200 mV). In chloride solutions, the DC potential variation is less significant at high AC density (higher than 1000 A·m^−2^). Results were confirmed in [39]: except for carbon steel in soil-simulating solution (1200 ppm SO_4_^2−^ (Na_2_SO_4_) + 200 ppm Cl^−^ (CaCl_2_·2H_2_O) [16]), the corrosion potential of galvanized steel, copper and carbon steel in different environmental conditions decreases with increasing AC density. Authors investigated the effects of AC density on anodic and cathodic overvoltages: AC has a significant effect on the kinetics parameters, with a decrease of overvoltages and increase of exchange current density of anodic and cathodic processes [39,40]. Nevertheless, in these papers some inconsistencies were observed between the experimental tests and the results expected from mathematical models based on the asymmetry of anodic and cathodic reactions. Li et al. [41] and Wang et al. [42] have measured a lowering of free corrosion potential on X70 and X80 carbon steel samples at various AC densities in simulated soil solution and 3.5% sodium chloride solution. Potential shift in both environments is about 0.2 V at 300 A·m^−2^ AC density. Moreover, authors investigated the kinetic effect of AC interference on anodic and cathodic overvoltages, measuring a variation of anodic and cathodic Tafel slope and their ratio in the presence of AC. Zhang et al. [43] proposed a nonlinear model (an electrical equivalent model considering the anodic and cathodic reactions under activation control) for the investigation of AC interference effect on corrosion potential and corrosion rate. Results show that the expected variation of the free corrosion potential depends on the AC peak potential, as expected, and on the ratio of the anodic and cathodic Tafel slope (*r* = βa/βc). When *r* = 1 (symmetry of anodic and cathodic overvoltages), no DC potential variations are predicted by the model. For *r* > 1, a positive (anodic) potential shift is expected, while for *r* < 1 the DC potential lowers as AC peak potential increases. The latter covers the electrochemical condition of active carbon steel in soil or waters where the cathodic processes (oxygen reduction and/or hydrogen evolution) have a higher Tafel slope than that of steel dissolution. These data are consistent with the observations made in [44] and in previous works [45,46,47].

#### 2.6.2. Positive Shift of Potential

In 2012, He et al. [24] report that the average corrosion potential of X65 steel in loam soil moves to more positive values by increasing AC density from 5 to 150 A·m^−2^. At 150 A·m^−2^, a positive shift of about 200 mV has been measured with respect to the condition without interference. Xu et al. [48] examined the effect of AC (60 Hz frequency) on 16Mn steel potential in a simulated soil solution by means of real-time AC/DC signal acquisition. AC moves corrosion potential negatively at an AC density lower than 400 A·m^−2^, while at higher AC density, the DC potential variation with respect to the absence of AC interference is positive. In a recent work, Wu et al. [49] reported polarization curves of X70 steel tested at AC densities up to 100 A·m^−2^ in simulated seawater. The presence of AC has a strong effect on the polarization curves with a general shift toward higher current density and a positive (anodic) variation of the zero-current potential, i.e., the free corrosion potential. Nevertheless, as the AC density was raised from 10 to 100 A·m^−2^, the corrosion current density and the free corrosion potential roughly remained constant.

### 2.7. Effect on DC Potential (Cathodic Protection Condition)

The potential measurement is affected by AC interference, even if CP is applied. Several authors have investigated in the last decades the effect of AC on IR-free potential. Bolzoni et al. [38] investigated the influence of AC interference on carbon steel in CP condition in different environments (sulfate and chloride aqueous solutions, with or without oxygen). In the presence of cathodic polarization, the potential trend depends on DC density: at 0.1 A·m^−2^, the DC potential is lowered after AC application; conversely, at 1 and 10 A·m^−2^ DC density, the DC potential increases as the AC density increases. In 2008 [50], and later in 2010 [23], Ormellese et al. reported the measurements of IR-free potential of carbon steel specimens exposed for about four months to a soil-simulating solution. DC and AC density were in the range 0.1–10 and 10–500 A·m^−2^, respectively. The increment of potential is not significant at 10 A·m^−2^ AC, while it is about 0.1 and 0.2 V at 100 and 200 A·m^−2^, respectively. The effect of AC density on IR-free potential is more pronounced at high DC density [23].

**Figure 2:**
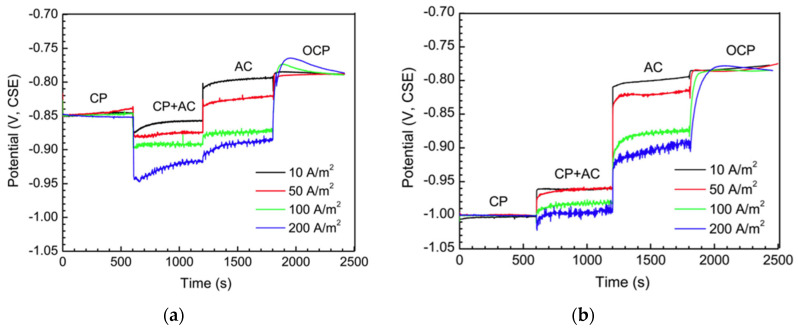
Effect of AC density on DC potential of X65 steel in near-neutral pH bicarbonate solution in cathodic protection condition: (**a**) −0.85 V vs. CSE, (**b**) −1 V vs. CSE [54].

**Figure 3:**
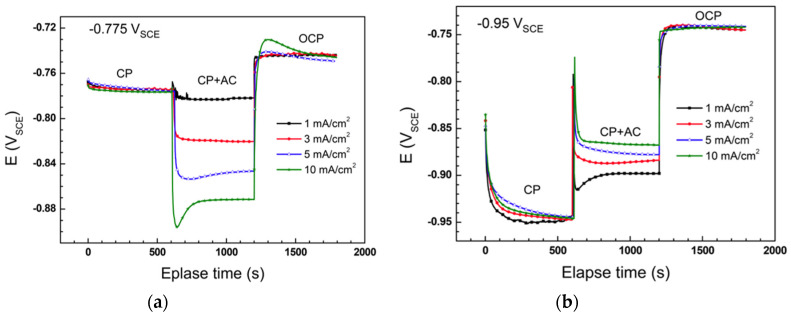
Effect of AC density on DC potential of X70 steel in near-neutral pH bicarbonate solution in cathodic protection condition: (**a**) −0.775 V vs. saturated calomel electrode (SCE) (−0.850 V vs. CSE) (**b**) −0.95 V vs. SCE (−1.02 V vs. CSE). 1 mA·cm^−2^ corresponds to 10 A·m^−2^ [55].

Xu et al. [51,52] investigated the effects of AC on the CP potential reading of a 16Mn pipeline steel in a simulated soil solution. At −0.85 V vs. SCE (maintained in a galvanostatic way, SCE—saturated calomel electrode), AC moves DC potential negatively. Furthermore, the higher the AC density, the more negative the DC potential is. Conversely at −1 V vs. SCE, AC shifts potential in the positive direction. Similar observations were reported by Kuang et al. [53,54]: the DC potential of X65 steel in near-neutral pH bicarbonate solution is shifted negatively by AC at −0.85 V vs. CSE, but positively shifted by AC under the CP of −1 V vs. CSE (Figure 2). Nevertheless, differently to what can be expected, the potential variation reported is higher at smaller AC density (Figure 2b). When the applied CP level was −0.925 V vs. CSE (data not shown), the DC potential becomes more positive at low AC densities of 10 and 50 A·m^−2^, while it decreases with 100 A·m^−2^ AC density. Recently, Wang et al. [55] reported similar conclusions for X70 steel in near-neutral bicarbonate solution: at −0.775 V vs. SCE, the DC potential is shifted negatively consequently to the application of AC, while at −0.95 V vs. SCE and −1.2 V vs. SCE, an increase of DC potential is measured (Figure 3). In this case, the potential variation is proportional to AC density.

Generally, it can be concluded that there is good agreement on the increase of DC potential in the presence of AC, although a negative shift is measured at small CP current density.

## 3. Acceptable AC Interference Levels—Protection Criteria

**Figure 4:**
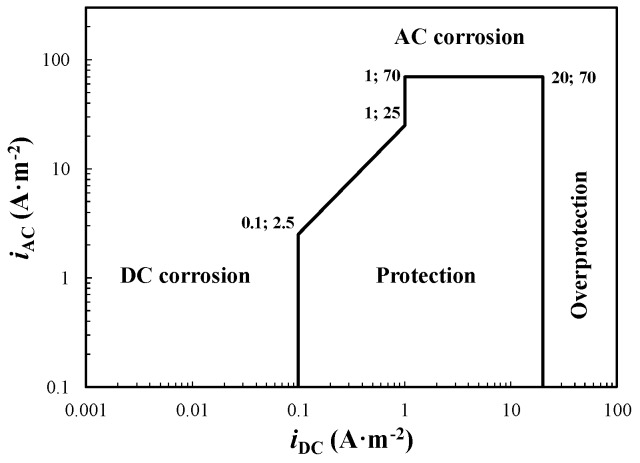
Acceptable AC interference levels based on current densities criterion according to Kajiyama et al. (adapted from [56]).

There is large agreement that corrosion can occur on AC interfered carbon steel structures that fully match the CP potential criterion (*E* < *E*_prot_) defined by ISO 15589-1 [2]. Much effort has been made in the last decades in order to define acceptable AC interference levels for carbon steel under CP condition. Kajiyama et al. [56,57,58,59] proposed a CP criterion based on the ratio between DC and AC densities, measured by means of corrosion coupons. The criterion can be summarized as follows (Figure 4):

- if 0.1 A·m^−2^ ≤ *i*_DC_ < 1 A·m^−2^, then *i*_AC_/*i*_DC_ < 25,
- if 1 A·m^−2^ ≤ *i*_DC_ ≤ 20 A·m^−2^, then *i*_AC_ < 70 A·m^−2^.

Accordingly, the maximum AC density depends on the CP level: at higher DC densities (i.e., more negative potential), a higher AC density can be tolerated. Even if the criterion has been applied successfully to some case studied [58], some authors recognized a greater AC corrosion risk at higher DC density differently to this criterion, as discussed later. Moreover, this criterion does not consider directly the value of the measured potential.

**Figure 5:**
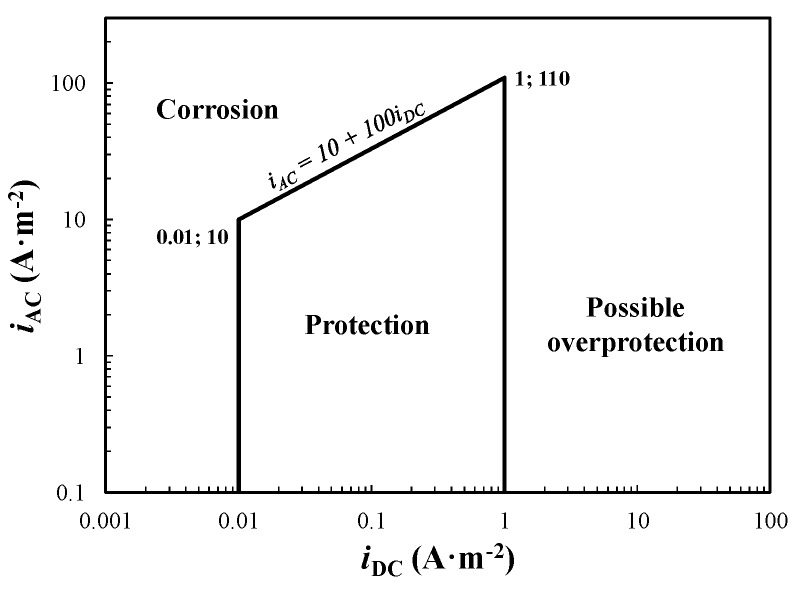
Acceptable AC interference levels based on current densities criterion according to He et al. (adapted from [24]).

In 2012, He et al. [24] reported a similar approach based on current densities: the AC density threshold increases linearly with CP current density. The criterion (Figure 5) suggests there is not corrosion risk if *i*_AC_ < 10 + 100·*i*_DC_ (with *i*_DC_ ≥ 0.01 A·m^−2^). Comparing the two criteria, the latter (Figure 5) is less conservative at a DC density lower than 1 A·m^−2^ and does not take into account AC corrosion at greater DC densities. For instance, at 0.1 A·m^−2^ DC density, the maximum allowed AC density is 2.5 and 20 A·m^−2^, considering the corrosion criterion of Figure 4 and Figure 5, respectively.

**Figure 6:**
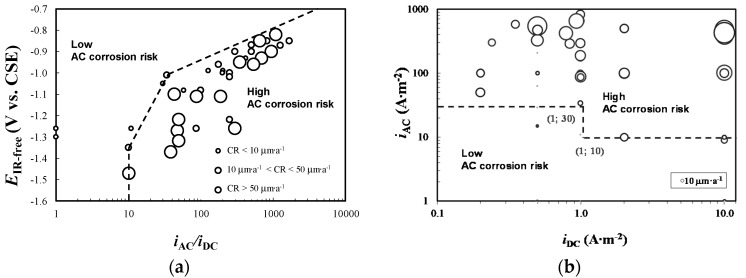
AC corrosion protection criterion as proposed by Ormellese et al.: (**a**) AC corrosion risk diagram based on IR-free potential and current densities ratio, (**b**) protection criterion based on AC and DC (i.e., cathodic protection—CP) current densities.

The effect of potential has been investigated by Ormellese et al. in [50] and later in [23,26]. The authors propose corrosion maps based on corrosion rate data evaluated using a weight loss test of carbon steel specimens exposed to a soil simulation environment, with varying AC interference and CP levels. Two AC corrosion risk regions are defined, low and high, for corrosion rates lower or greater than 10 μm·a^−1^, respectively. Corrosion risk increases by increasing the *i*_AC_*/i*_DC_ ratio (Figure 6a): corrosion protection is achieved up to a maximum value of the *i*_AC_*/i*_DC_ ratio, which decreases as the IR-free potential becomes more negative. Differently from the criteria discussed previously, in overprotection condition a few A·m^−2^ of AC density (ranging from 5 to 20 A·m^−2^, depending on potential) provokes corrosion of overprotected carbon steel. The authors proposed the following criterion (Figure 6b):

- if 0.1 A·m^−2^ ≤ *i*_DC_ < 1 A·m^−2^, then *i*_AC_ < 30 A·m^−2^,
- if 1 A·m^−2^ ≤ *i*_DC_ ≤ 10 A·m^−2^, then *i*_AC_ < 10 A·m^−2^.

The −0.85 V vs. CSE criterion is not always safe in the presence of AC interference; in overprotection condition (*E*_IR-free_ < −1.2 V vs. CSE), severe AC corrosion could occur.

Fu et al. [60] proposed a criterion based on both potential and current densities: potentials more positive than −0.95 V vs. CSE are considered not safe in the presence of AC. The maximum acceptable AC density increases as the potential becomes more negative: at −0.95 V vs. CSE, the maximum AC density is 20 A·m^−2^, while at −1.05 V vs. CSE, the threshold increases up to 100 A·m^−2^.

**Figure 7:**
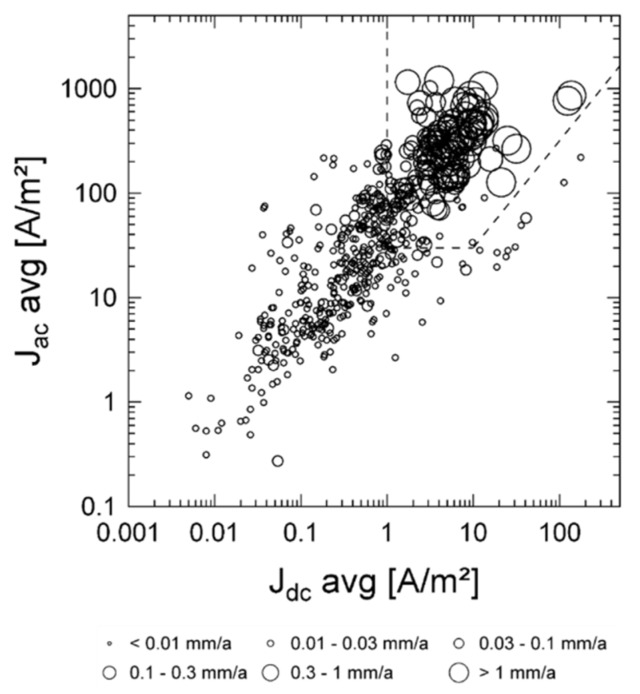
Protection criterion based on AC and DC densities as proposed by Büchler [61]. The region confined by the dotted lines corresponds to very severe AC corrosion and must be avoided.

Büchler in 2012 [61] and previously in 2009 [22] investigated new protection criteria based on laboratory and field investigations. For current density average values measured by means of on-site coupons, one of the criteria below must be met (Figure 7):

- *i*_AC_ < 30 A·m^−2^;
- *i*_DC_ < 1 A·m^−2^;
- *i*_AC_/*i*_DC_ < 3.

**Figure 8:**
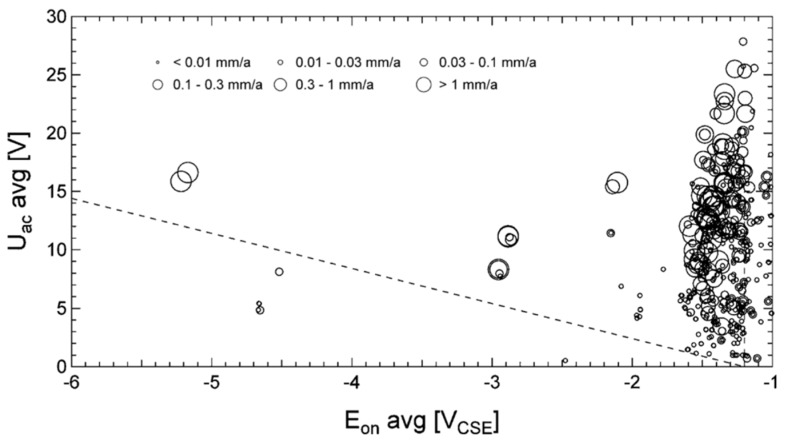
Protection criterion based on AC voltage and ON potential, as proposed by Büchler [61]. The region below the dotted lines corresponds to acceptable AC interference conditions.

Nevertheless, the author [61] stated that the measurement with coupons is fraught with problems, since the obtained results are affected by coupon geometry and local soil conditions. The use of ON potential, *E*_ON_, and AC voltage (*V*_AC_, indicated by the authors as *U*_AC_) is therefore suggested (Figure 8). For ON potential, one of the following criteria must be met:

- average *V*_AC_ < 15 V and average *E*_ON_ more positive than −1.2 V vs. CSE;
- *V*_AC_ < 3·(|*E*_ON_| − 1.2) where *E*_ON_ is in V vs. CSE and *E*_ON_ < −1.2 V vs. CSE.

In 2015, these efforts brought to the AC corrosion protection criteria of the ISO 18086 standard [4] for buried carbon steel in CP condition:

- As a first step, the AC voltage on the pipeline should be decreased below 15 V r.m.s. The AC voltage is measured as an average over a representative time (e.g., 24 h) with respect to a reference electrode located in remote position;
- As a second step, AC corrosion mitigation is achieved by matching the CP protection potentials defined in ISO 15589-1 [2] and
  — maintaining the AC density (*i*_AC_) lower than 30 A·m^−2^ on a 1 cm^2^ coupon or probe over a representative time (e.g., 24 h), or
  — maintaining the average cathodic current density lower than 1 A·m^−2^ on a 1 cm^2^ coupon or probe over a representative time (e.g., 24 h), if AC density is higher than 30 A·m^−2^, or
  — maintaining the ratio between AC and DC densities (*i*_AC_*/i*_DC_) less than 3 over a representative time (e.g., 24 h).

**Figure 9:**
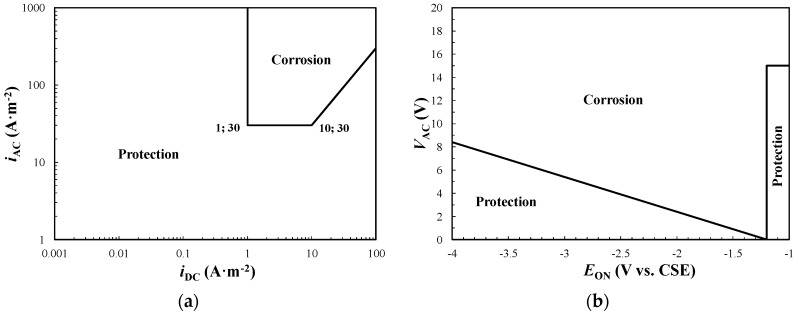
Graphical representation of the AC protection criteria reported on ISO 18086 [4]: (**a**) *i*_AC_ vs. *i*_DC_, (**b**) *V*_AC_ vs. *E*_ON_.

In Annex E (informative), the standard reports other criteria that have been used in the presence of AC. These criteria have been derived from Büchler’s work [61] and are based on AC voltage, ON potential and current densities, as discussed. The protection criteria reported in the ISO 18086 standard are shown in Figure 9.

In 2018, Junker et al. [62] reported results of laboratory and field tests varying AC and DC levels and soil chemistry. Both laboratory and field data confirmed very high AC corrosion rates under excessive CP (> 1 A·m^−2^) and AC interference higher than 30 A·m^−2^. Moreover, they recognized in the spread resistance of a coating defect a highly dynamic parameter under AC and DC influence. The investigations illustrate that the chemical environment alters the AC and DC density limits for AC corrosion, however the present limits of ISO 18086 constitute a safe strategy in most environments.

**Figure 10:**
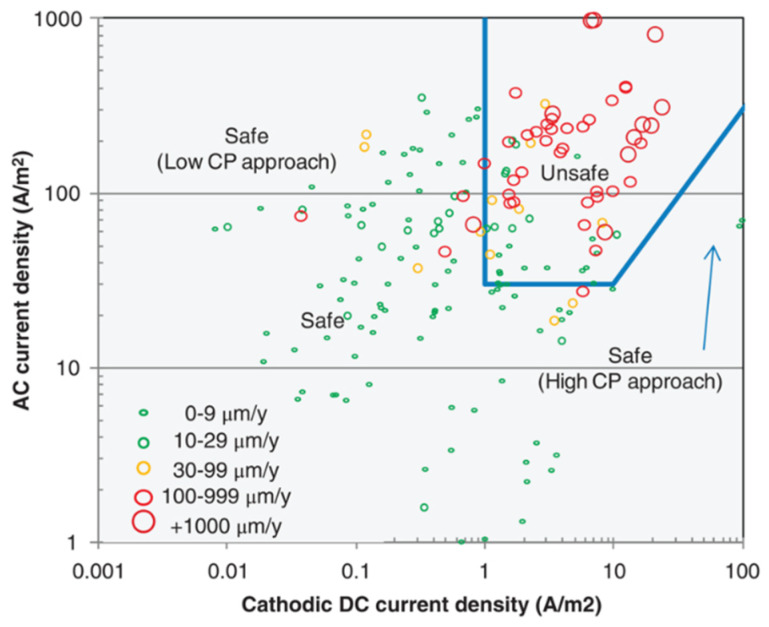
AC corrosion rate measured on corrosion coupons as reported by Nielsen [63]. Data are compared with the AC protection criteria reported in ISO 18086.

Figure 10 reports the AC corrosion rate measured on corrosion coupons with varying AC and DC density, as reported by Nielsen [63]. Data are overlapped to the AC protection criteria reported in ISO 18086.

It can be concluded that overprotection (namely *E*_IR-free_ lowers than −1.2 V vs. CSE [2] or *i*_DC_ > 1 A·m^−2^) is the most dangerous condition in the presence of AC interference. At “high” CP levels, the maximum tolerable AC density is 30 A·m^−2^. Below 1 A·m^−2^ DC density, the AC corrosion likelihood decreases. Nevertheless, some doubts are revealed regarding the inexistence of an AC density threshold at “low” CP condition (*i*_DC_ < 1 A·m^−2^).

## 4. AC Corrosion Mechanism

Numerous theories and models have been proposed on the AC corrosion mechanism of carbon steel. Some models referring to carbon steel structures in free corrosion conditions as well as in the presence of CP are discussed hereafter.

### 4.1. Effect of Anodic and Cathodic AC Half-Wave on Metal Dissolution

**Figure 11:**
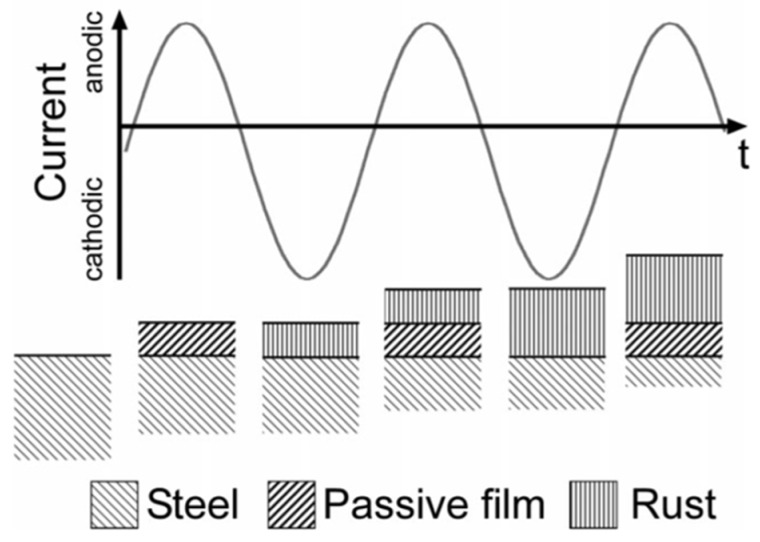
Schematic representation of AC corrosion mechanism according to Büchler et al. [61].

Büchler et al. [22,61] proposed a corrosion mechanism based on thermodynamic and kinetic considerations on the reactions involved during AC interference on cathodically protected carbon steel. When an AC voltage is present, current will flow through the coating defects exposed to soil. If the pH value is sufficiently high (above 10, as in CP condition), during the anodic half-cycle, steel oxidation occurs, promoting the formation of a passive film. During the cathodic half-wave, the passive film is electrochemically destroyed and converted in porous rust. In the successive anodic cycle, the passive film is reformed under the non-protective rust layer. Moreover, the Fe^2+^ present in the rust layer is oxidized to Fe^3+^ (Fe^2+^ → Fe^3+^ + e^−^). In the subsequent cathodic cycle, the dissolution of the passive film will increase the volume of porous rust. Hence, every AC cycle results in the oxidation of the metal with a significant metal loss in the long term (Figure 11). A simplified description of this mechanism is reported also in the ISO 18086 standard [4].

### 4.2. The Alkalization Mechanism and the Effect of Spread Resistance

Nielsen et al. [64,65,66] proposed the “alkalization model” of AC-induced corrosion of carbon steel under CP condition. The model is based on the combination of two effects: (1) the alkalization of the metal-to-electrolyte interface of overprotected carbon steel, and (2) potential oscillations across the immunity, the passive and the high-pH corrosion domain of the iron potential-pH diagram.

**Figure 12:**
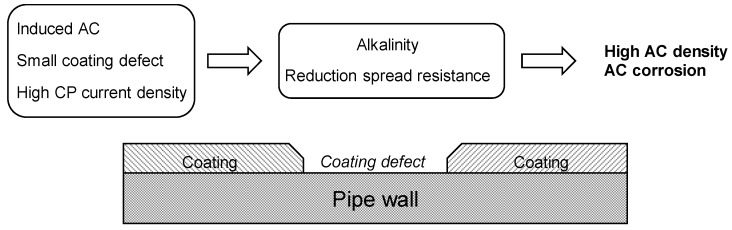
Schematic representation of the AC corrosion mechanism of carbon steel under CP condition, as proposed by Nielsen et al. [63,64,65,66].

As known, the presence of a cathodic current on the metal surface under CP condition is beneficial because it promotes the reduction (or zeroing) of the corrosion rate and the increase of alkalinity due to the accumulation of hydroxyl ions (OH^−^) close to coating defect. The pH increase is proportional to the cathodic current density and depends on the diffusion and electrical migration of ions towards and from the metal surface. In overprotection condition, namely IR-free potential more negative than −1.2 V vs. CSE, the high cathodic current density (in the order of a few A·m^−2^) can promote a significant increase of pH up to 13 or higher. According to the Pourbaix diagram, at elevated pH the potential oscillations caused by AC interference could lead to periodic entry in the high-pH corrosion region with formation of dissolved HFeO_2_^−^ ions. The authors report the presence of an “incubation time” defined as the period to reach a critical pH (close to 14) at the metal-to-soil interface, with a significant lowering of the spread resistance and increase of AC density due to depolarization effects of the AC (Figure 12). Then, potential oscillations could cause corrosion due to different time constants associated to iron dissolution (fast) and the formation of a passive film (slower). Accordingly, AC corrosion of carbon steel in CP condition cannot be reduced by adding a surplus of cathodic current, as in the case of DC corrosion phenomena, but by avoiding high DC densities and the overprotection condition, in agreement with the protection criteria of the ISO standard [4].

**Figure 13:**
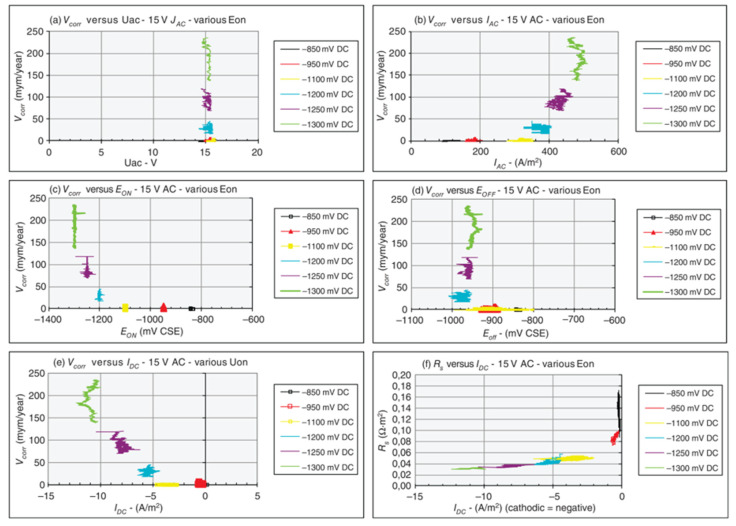
Example of corrosion rate vs. various electrical parameters, as reported by Nielsen [63].

This “vicious circle” is supported by the data shown in Figure 13a–f [63], which illustrates experimental results in a laboratory soil box environment in purified quartz sand. At a constant AC voltage (15 V), six different levels of CP (ON potential) were applied for some weeks to monitor the corrosion rate of an electrical resistance probe and various electrical parameters. At a fixed AC voltage, the corrosion rate depends strongly on the CP level (ON potential), and therefore, AC voltage alone cannot be considered a valid indicator of AC corrosion risk. Despite the constant AC voltage, the AC density varies from about 100 A·m^−2^ at low CP levels up to 500 A·m^−2^ at higher CP levels. Figure 13e shows corrosion rate as a function of cathodic current density: the corrosion rate increases with increasing CP current density, in agreement with the proposed mechanism. At the same time, the spread resistance is strongly influenced by the DC density with the consequent increase of AC density and corrosion rate (Figure 13f).

The crucial role of soil chemical composition, pH and spread resistance was carefully investigated by Junker et al. [62,67,68,69]. The spread resistance is identified as a key parameter, controlling the current densities, and is highly influenced by the formation of calcareous deposits or corrosion products. At high CP density, AC corrosion of an ER probe element is associated with strong alkalization of the electrolyte and consequently dense calcareous deposit formation. The calcareous deposit dramatically increases the spread resistance and reduces the AC and DC densities. Corrosion decreases, but only as long as the calcareous deposit is stable and fully covering the surface. Due to brittle fracture or a ‘flake of’ mechanism of the scale (probably provoked by hydrogen evolution at low potentials), the spread resistance suddenly decreases, causing an increase of current densities and AC corrosion. The cathodic reactions on the re-exposed probe surface will restart the alkalization and precipitation of calcareous deposits and with time (days) the corrosion stops again. This causes a cyclic variation of spread resistance, current densities and corrosion rate. The detailed chemical investigation of stone hard soil formed on cathodically protected pipeline under AC interference is reported in [68].

The effect of spread resistance on AC corrosion has been also documented by Nielsen and Cohn [70] with the help of an electrical equivalent circuit analysis that represents the impedances existing between the pipe and remote earth. AC and DC sources impose a DC and AC voltage on the pipe: the simulated AC source is the HVTL (High Voltage Transmission Line), whereas the DC source represents the CP system. The authors consider static and dynamic elements. Static elements, namely spread resistance and charge transfer resistance, are defined as elements where impedance is frequency independent. Conversely, dynamic elements are frequency dependent: these include the double layer capacitance and diffusion elements. Because of the low impedance of the capacitance, the spread resistance is the dominant impedance element at 50–60 Hz frequency and plays a key role in controlling the AC corrosion process.

### 4.3. Effect of AC on Anodic and Cathodic Overvoltages

**Figure 14:**
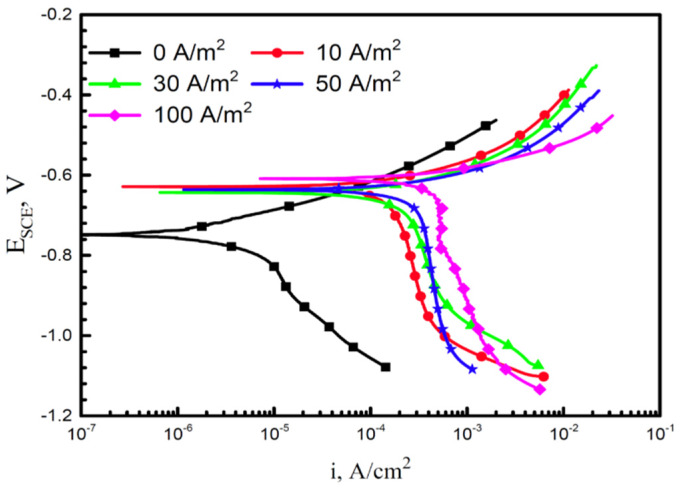
Polarization curves on X70 steel tested at various AC densities in simulated seawater [49].

The increase of corrosion rate in the presence of AC has been explained by some authors using the effect of AC on anodic and cathodic overvoltages [39,46,49,65,71,72]. Goidanich et al. [39] investigated by means of laboratory tests the influence of AC on kinetic characteristics of carbon steel, galvanized steel, copper and zinc under different experimental conditions. Results showed that AC has a significant influence on kinetic parameters, such as Tafel slope and exchange current density, and on corrosion and equilibrium potential. The authors proposed a “mixed” corrosion mechanism, with a general decrease of overvoltages and increase of exchange current density in the presence of AC. This effect could be related to the local rise in temperature, associated with high AC densities, as reported in [16]. In a recent work, Wu et al. [49] reported polarization curves of X70 steel tested with varying AC densities (up to 100 A·m^−2^) in simulated seawater (Figure 14). AC shifts toward higher current density in the polarization curves, promoting both the anodic and cathodic processes.

As discussed previously, some authors [43,44,45,46,47] proposed theoretical models based on the fundamental thermodynamic and kinetic laws of corrosion in order to investigate the effect of AC interference on DC potential and corrosion rate. Accordingly, the sensitivity of the corroding system is influenced by the ratio of the anodic-to-cathodic Tafel slope (*r* = β_a_/β_c_). The effect of *r* on DC potential variation has already been discussed in Section 2.6. Considering a corrosion system under activation control, Lalvani and Lin [45] proposed an analytical solution for the relationship between corrosion rate and voltage peak amplitude. In a revised model [46], the authors introduced the effect of the double layer capacity, without considering the resistance of the electrolyte. The model indicates that corrosion current increases with voltage peak for all values of *r*, while the potential shift depends strongly on the anodic and cathodic characteristic curves, i.e., on the ratio between the anodic and cathodic Tafel slope. Potentiodynamic polarization curves were obtained using the revised model; nevertheless, these approaches predict that corrosion current and corrosion potential are independent of the frequency of the AC signal, differently to what is observed.

**Figure 15:**
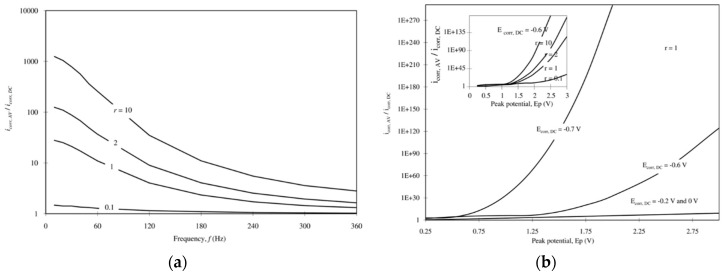
A nonlinear model for corrosion of metals subjected to AC corrosion [43]: (**a**) dimensionless corrosion current vs. frequency, (**b**) dimensionless corrosion current vs. peak potential and DC corrosion potential. Legend: *E*_corr,DC_ = corrosion potential in the absence of AC (V vs. SCE); *E*_p_ = AC peak potential (V vs. SCE); *r* = β_a_/β_c_.

The model was improved in 2008 [43,44]; the authors considered three elements in an electrical equivalent circuit of a metal subjected to an induced AC voltage: the polarization impedance, the double layer capacitance and the electrolyte resistance. The model shows that the corrosion current increases as the frequency of the AC signal decreases (Figure 15a), in agreement with experimental observations, and by increasing the peak potential. Moreover, the model shows that corrosion current increases by decreasing the DC corrosion potential. For instance, by decreasing the DC corrosion potential from −0.6 to −0.7 V vs. SCE at a peak potential of 1.25 V vs. SCE, the corrosion rate increases several orders of magnitude (Figure 15b); an increase of DC corrosion potential from −0.2 to 0.0 V does not result in a further reduction of corrosion current [43].

Recently, Ibrahim et al. [73,74,75] proposed a theoretical approach (Part 1, 2, 3) to evaluate the effect of double layer capacitance and electrolyte resistance on corrosion current density and potential shift. The authors stated that corrosion rate enhancement is due to the faradaic rectification as a consequence of the nonlinear current-potential relationship [73].

### 4.4. Breakdown of the Passive Film and High-pH Corrosion

Recently, Brenna et al. [76,77] proposed a two-step AC corrosion mechanism. In the first step, AC causes the weakening of the passive film formed under CP condition on carbon steel, due to electromechanical stresses. Electrostriction appears to be a convincing explanation of the passive film breakdown mechanism, because of the presence of high alternating electric field (in the order of 10^6^ V·cm^−1^ [78]) across the passive film [77]. The effect of AC on passive condition has been documented for carbon steel in alkaline solution or concrete and for stainless steel in neutral solution [79,80,81,82,83,84,85]: AC causes localized corrosion of passive metals with a decrease of corrosion resistance. Mechanical failure of the film can result from high electromechanical stresses (electrostriction pressure) generated by the presence of an electric field across the film and by the interfacial tension, which is not negligible as a result of the thin thickness of the oxide.

After film breakdown, high-pH chemical corrosion (i.e., potential independent) occurs in the overprotection condition because of the high cathodic current density supplied to the metal. According to the Pourbaix diagram of iron, high-pH corrosion can occur with formation of di-hypo ferrite ions (HFeO_2_^−^). This mechanism can also explain the unexpected corrosion of the metal below its equilibrium potential. Indeed, CP electrons are involved in the cathodic process, which depends on the potential assumed by the metal. Thus, below the equilibrium potential, the applied cathodic current makes electrons available to the metal; therefore, no anodic electrochemical reactions can take place. Consequently, if no oxidation reaction takes place, such as iron ion production, this can occur only through chemical corrosion, which is not potential dependent, or, in other words, is not influenced by the electrons made available otherwise [86].

## 5. AC Corrosion Monitoring

According to ISO 18086 [4], AC voltage should be measured with respect to remote earth at test posts during the general and detailed assessment of CP effectiveness, as defined in ISO 15589-1. Additional measurements should be carried out at sites where AC corrosion risk is suspected, e.g., areas where the soil resistivity is low (lower than 25 Ω·m), areas with highest AC interference levels, areas where AC corrosion has previously taken place and areas where local DC polarization conditions can favor AC corrosion, as high levels of CP.

The measurement of AC and DC densities should be carried out by means of coupons or probes installed in the same soil or backfill as the pipeline itself. The measurements with respect to the criteria defined in ISO 18086 have to be carried out on a 1 cm^2^ coupon surface area. Coupon or probe currents can be measured by the voltage drop across a series resistor; the value of the resistance should be sufficiently low to avoid disturbance of the system. For field measurements, a typical value is 10 Ω for a 1 cm^2^ coupon.

For corrosion rate measurements, weight loss measurements, perforation measurements or electrical resistance (ER) measurements can be applied. Weight loss measurements require installation of pre-weighed coupons. After some time of operation (months to years), the coupon is excavated and weight loss rate is determined. The main drawback of this measurement is that the coupon provides no information until the excavation. Perforation measurements are made on special perforation probes: a signal is generated when the corrosion process has perforated the wall thickness of the coupon. In this case, information about the maximum (localized) corrosion depth is available without excavating the coupon; the primary disadvantage is that this information is not available until the coupon is perforated. Electrical resistance measurements require the installation of electrical resistance probes (ER probes). Corrosion is detected by the increase of the electrical resistance of the coupon when corrosion progressively decreases its thickness [64,87,88].

## 6. Conclusions

This paper deals with a narrative literature review on AC corrosion assessment, protection criteria and corrosion mechanisms for buried carbon steel structures in CP condition. Main conclusions can be summarized as follows:

- The assessment of AC corrosion likelihood should be based on the measurement of AC and DC related parameters. The AC interference level is evaluated by AC remote voltage and AC density, while the CP level is assessed by DC density and potential measurements;
- AC and DC densities should be measured by means of a corrosion coupon (1 cm^2^ area) connected to the structure in CP condition; IR-free potential is considered more accurate than ON potential, because it does not contain the ohmic drop contribution in soil;
- There is general agreement that the DC potential of carbon steel in CP condition increases in the presence of AC interference, although a negative shift is measured at small DC density. Conversely, in free corrosion condition, i.e., without CP, the potential decreases as the AC density increases;
- Overprotection (namely *E*_IR-free_ < −1.2 V vs. CSE) is the most dangerous condition in the presence of AC interference. At “high” CP levels, the maximum tolerable AC density is 30 A·m^−2^. Below 1 A·m^−2^ DC density, the AC corrosion likelihood decreases. Nevertheless, some doubts are revealed regarding the inexistence of the criterion reported in the ISO 18086 standard of an AC density threshold at “low” CP condition (*i*_DC_ < 1 A·m^−2^);
- The higher AC corrosion likelihood at high CP levels could be explained by a corrosion mechanism that involves both the AC and DC levels:
  ૦ At high DC density, a strong alkalization of the electrolyte close to the coating defect occurs with formation of a passive film and deposits (as a calcareous deposit) on carbon steel. Soil chemical composition, pH and spread resistance at the coating defects seem to have a crucial role, controlling the local AC and DC densities;
  ૦ AC interference provokes a weakening of the passive condition due to an effect on anodic and cathodic overvoltages; moreover, the scale formed in CP condition is not stable in the presence of AC due to potential oscillations that could break the protective layer;
  ૦ High-pH corrosion occurs with localized corrosion attacks; chemical corrosion (i.e., potential independent) with formation of di-hypo ferrite ions (HFeO_2_^−^) is a possible explanation for the occurrence of corrosion at low potentials.
